# Physiology and Psychology of Vision and Its Disorders: A Review

**Published:** 2014

**Authors:** Marilita M Moschos

**Affiliations:** Department of Ophthalmology, University of Athens, Greece

**Keywords:** Age-related Macular Degeneration, Depression, Illusion, Vision Loss

## Abstract

The purpose of this review is to bring together to the physiology and psychology of vision and to analyze, based on our own data and on the available literature, the relationship between sight loss and individual reactions. As recent treatments for depression are often effective and have few side-effects, ophthalmologists should consider referral for treatment of depression in patients suffering from vision impairment. For this reason, vision rehabilitation should be more readily available and recommended.

## INTRODUCTION

The question “how do we see?” may be analyzed from many viewpoints and from many philosophies. Having come up to it in one way, we may, by turnaround of vantage points, come to see it very differently. By the brain having rather little to do, considering the classical account, perception is passive pick-up of information from the world. Another, the opposite view is that the brain or mind is highly active, constructing perceptions from hardly adequate information obtained from the senses. On this view, illusions of many kinds take on a remarkable significance. Illusions have generally been written off as annoying and sometimes dangerous, but trivial.

Why and how do we experience colors, shapes, sounds, and pains? What and why is consciousness? Thirty, even fifty years ago, these questions were generally seen as being beyond the science.

Then again, vision disorders are a major public health problem, because they cause disability, suffering, and loss of productivity. It has been acknowledged for long that vision loss may generate various degrees of psychic suffering, undoubtedly greater than the distress resulting from other forms of sensory impairment.

The aim of this review is to study the relationship between the physiology and psychology of vision and to analyze, based on our own data and those from the available literature, the relationship between sight loss and individual reactions.

## PHYSIOLOGY OF VISION

We are so familiar with seeing that it takes a leap of imagination to realize that there are problems to be elucidated. In fact, we perceive distorted reversed images in the eyes, and we see detached solid objects in surrounding space. From the patterns resulting of stimulation of the retina, we perceive the world of objects.

The eye is often likened to a camera, which far from correct. What the eyes do is inputting the brain with the information hinted into neural activity, which by their code and the patterns of brain activity, represents objects. Equivalents may be spotted with written language: the letters and words of a text have certain meanings to those who know the language. They affect the reader’s brain appropriately, but they are not pictures. When we look at something, the pattern of neural activity represents the object and to the brain, it is another object. No internal picture is involved (1).

Each part of the human eye is an extremely specialized structure and the most important optical instrument. Here lies the focusing lens, giving a miniature-inverted image to an incredibly dense mosaic of light-sensitive receptors, which convert the patterns of light energy into the language the brain can read -- chains of electrical impulses.

The neural system responsible for vision starts with the retinas. These are essentially outgrowths of the brain, comprising typical brain cells as well as specialized light-sensitive detectors, the rods and cones. The cones function in daylight conditions and give color vision. The rods function under low illumination and give vision only of shades of gray. In the human retina, there are very few cones near the edge of the retina, where there is no color vision.

Each retina is divided as reads starting from the center -- the optic nerve fibers from the inner (nasal) halves crossing at the chiasma whereas fibers from the outer halves do not cross. So, the images conveyed from the e.g. right half of each retina are embodied on the right side of the occipital cortex and vice versa. The visual region of the brain is known as the area striata.

## LIGHT STIMULUS

We need the light to see. Plato thought of vision as preventing the light to enter, but rather to particles blast out of the eyes, spraying surrounding objects.

For the last 300 years, there have been two rival theories of the nature of light. Isaac Newton (1642-1727) argued that light must be a sequence of particles, while Christiaan Huygens (1629-93) argued that it must be throbs traveling through an all pervading medium - the Aether - which he thought of as small elastic balls in contact with each other. Any disturbance, he suggested, would be carried in all directions through the packed spheres as a wave, and this wave is light.

We consider the light consisting of packets of energy - quanta - combining characteristics of corpuscles and waves.

Light is a narrow strip of the entire electromagnetic spectrum, which includes radio waves, infrared, ultra violet and X-rays. This means that only a very narrow band of these frequencies, less than an octave in width, stimulates the eye to give vision and color. Considering it this way, we are almost blind.

Because of the finite velocity of light, and the delay in nervous messages reaching the brain, we always sense the past. Our perception of the sun is over 8 minutes late: all we know of the furthest object visible to the unaided eye (the Andromeda nebula) is so out-of-date that we see it as it was a million years before men appeared on Earth.

Almost every living thing is sensitive to light. Plants accept the energy of light, some moving to follow the sun. Animals use light, shadows and images to avoid danger and to seek their prey (2). Response to light is found even in one-celled animals. In higher forms, we find specially adapted cells to serve as receptors sensitive to light.

Among the most curious eyes in the whole of nature is that of a creature the size of a pin’s head, a copepod called Copilia quadrata. She (the males are dull) has a pair of image-forming eyes, which function as scanning eyes, like a mechanical television camera. Each eye contains two lenses: a large anterior lens and a smaller lens deep in the body, with an attached photoreceptor and a single optic nerve fiber conveying impulses to the central brain. Most of the eye lies deep within the body of the animal, which is extraordinary transparent. The second smaller lens and photoreceptor are in continual movement, across the image plane of the first large anterior lens (3).

## BRIGHTNESS (1-4)

Seeing brightness is an experience. Luminance is the intensity of physical energy of the light, which may be measured by various kinds of the photometer (4).

Another important distinction to be made is the color as a sensation and color as a wavelength of the light entering the eye. Light itself is not colored: it gives rise to sensations of brightness and color, but only in conjunction with a suitable eye and nervous system.

Brightness is a function not only of the intensity of light falling on a given region of the retina at a certain time, but also of the intensity of the light that the retina has been subject to in the recent past and of the intensities of light falling on other regions of the retina.

Brightness is also a function of color. If we shine lights of different colors but the same intensity into the eyes, the colors in the middle of the spectrum will look brighter than those at the ends. This is shown in figure, the curve being known as the spectral luminosity curve. This is of some practical importance for if a distress signal light is to be clearly visible, it should be of a color to which the eye is maximally sensitive. The cones are most susceptible to yellow, while the rods are most prone to green. The change of increasing intensity is known as the Purkinje effect (sometimes called the Purkinje shift, or dark adaptation).

## MOVEMENT

An essential psychological question is why the world remains stable when we move our eyes. The retinal images run across the receptors whenever we move our eyes, and yet we do not experience movement – the world does not, usually, spin round when we move our eyes. There are two neural systems for signaling movement, the image/retina and the eye/head movement systems. It seems that during normal eye movements their signals cancel each other out to give stability to the visual world (5).

Hermann von Helmholtz is the greatest figure in the experimental study of vision. Since his Physiological Optics, little has been added. According to his theory, known as the outflow theory the retinal movement signals are cancelled by central “outflow” signals from the brain commanding the eyes to move (6).

## VISUAL DISTORTIONS AND ILLUSIONS

Perception can go wrong in many ways. Most dramatic- an entire world may be created and mistaken for reality. This can happen in drug-induced states, or in mental disease. In addition to hallucinations, where experience totally departs from reality, people during “normal” circumstances may perceive surrounding objects in a distorted way (7-8).

Hallucinations are similar to dreams. They may be visual or involve any of the other senses. They may even combine several senses, when the impression of reality is overwhelming. Dreams and hallucinations have always excited wonder and sometimes more, as they have, in times, affected personal and political decisions (8).

To physiologists, dreams and hallucinations are due to spontaneous activity of the brain, unprovoked by the sensory stimuli. Brain tumors and the “aura” preceding epileptic seizures may give visual experiences. In these cases, the perceptual system is activated not by the normal signals from the photoreceptors, but by stimulation that is more central. Hallucinations occur in isolated people, when sensory stimulation is not existent, so that the brain can run wild and produce fantasies (7-8).

It is possible that this is as what happens in schizophrenia, when the external world and the individual make little contact, so he is effectively isolated (8).

Some simple figures are seen distorted. Part of the figure may appear 20% too long, or too short. Virtually all of us perceive these distortions and in the same directions for each figure. The best-known illusions, named according of their discoverers, mainly 19th century physicists and psychologists (9).

Muller-Lyer’s illusion, or arrow illusion – the outgoing fins looks longer than the figure with the ingoing fins, though it is, in fact, the same length (10).

Psychologists and physiologists have tried to explain the distortion illusions for the last 100 years, leaving any explanation as controversial, and the theories are the followings:

- The eye movement theory
- The limited acuity theory
- Physiological “confusion” theories
- The empathy theory.

The caryatids of Greek temples embody this idea in architecture. The idea is that the observer identifies himself with parts of the figure and that he becomes emotionally involved so that his vision is inaccurate, in a way emotion may distort an intellectual judgment.

- The pregnancy of “good-figure” theory
- The perspective theory.

An interesting observation among cultural differences is that in the western world, rooms are nearly always rectangular and many objects, such as boxes, have right-angled corners. Roads and railways present long parallel lines converging by perspective. People living in the western world, described as “rectangular” western culture, have a visual environment rich in perspective cues to distance. The people who standout as living in a nonperspective world are the Zulus (11) living in a world of “circular culture”, with round huts , plowing land in curves, with few straight lines or corners at all. They experience the arrow illusion only partly and are almost not affected by the illusion of distorted figures.

## LEARNING TO SEE

A most ancient question in psychology of vision is: “How do we come to know the world? Do we have to learn how to see?” There are many animals, which seem to know a lot about the world of objects before they experience it. Migrating birds use the pattern of the stars to guide them over bland oceans, even when they have never seen the sky.

Experimental psychology distinguishes between innate and learned responses: the former implying knowledge without previous experience, opposing to the latter, which build knowledge on observation. Animals, including insects, can respond appropriately to objects upon first encounter.

However, getting direct evidence from babies is difficult because they are poor with motor expression and produce no functional language. It was demonstrated that they have coordinated eye movements within a few weeks of birth. They also prefer solid objects (three-dimensional) to flat representations of the same objects; as a result, possibly they have some innate appreciation of the depth (12).

Touch puts forth important effects on vision -- during the early stages of adaptation; objects would tend to look suddenly normal when touched, and they would also look normal when they could not be touched without seeing. The conclusion of a wide variety of experiments on displacements, distortions and time shifts of retinal images is that it cannot be proved how babies have to learn how to see. However, we do have perceptual learning of various kinds even when adult. In animal and some human experiments, it is difficult to know whether the adaptation is perceptual or proprioceptive (12).

## THE POWER OF SEEING AND BELIEVING

The sense organs receive patterns of energy, but we seldom see mere patterns: we see objects. A pattern is a relatively meaningless arrangement of marks, but objects have a host of characteristics beyond their sensory features. They change and influence each other and have hidden aspects, which emerge under different conditions. We do not generally define objects by how they appear, but rather by their uses and their causal characteristics. The importance of relations in perceptions was studied by Albert Michotte at Louvain, who for many years investigated the perception of causality (13).

The perceptual system has been of biological significance for far longer than the calculating intellect. The regions of the cerebral cortex concerned with thought are comparatively juvenile. They are self-opinionated by comparison with the ancient regions of the brain giving survival by seeing. The perceptual system does not always agree with the rational thinking cortex. For the cortex educated by physics, the moon’s distance is 390000 km; to the visual brain it is a few hundred meters. Though in this example the intellectual cortical view is the correct one, the visual brain is never informed and we continue to see the moon as though it is located a few hundred meters away from the Earth (14).

The visual brain has its own logic and preferences, which are not yet understood by us cortically. Some objects look beautiful, others ugly, but we have no idea for all the theories, which have been put forward why this should be so. Knowledge of nonvisual characteristics affects how objects are seen. This is true even of people’s faces: a friend looks quite different from other people; a smile is not just a baring of teeth, but an invitation to share a joke.

A man born blind never learns to interpret facial expressions, though he can read a mood from the sound of a voice.

## VISION DISORDERS

Vision disorders are a major public health problem because they cause disability, suffering, and loss of productivity (15). Difficulties with vision occur among people of all ages, impact most disciplines within public health, and have broad health implications. The prevalence, type and effects of the disorders vary among the different age groups. Vision disorders result from developmental problems, uncoordinated growth of the elements of the eye, disease processes such as inflammation and degeneration, and other changes in the anatomy and physiology of the eye. These disorders affect individuals by reducing their visual acuity, visual fields, color vision, or stereopsis. Fortunately, most vision disorders can be treated, though not cured. At least 90% of all problems that people have with their eyes result from refractive errors, strabismus and amblyopia. Less than 10% of vision problems result from diseases, such as senile cataract, senile macular degeneration, diabetic retinopathy or glaucoma. In the population over 45 years old, virtually everyone has some vision disorder. (16)

Blindness is defined legally as visual acuity (VA) less than 20/200 or worse in the better eye with the best ophthalmic correction or visual fields less than 20 degrees in diameter. Blindness may be absolute with no light perception.

Global data on blindness suggest that cataract, refractive error and trachoma are the most important causes of blindness in developing countries whereas age-related macular degeneration is the leading cause in the US, and in established market economies (17, 18). The relationship between lower socioeconomic status and higher rate of blindness is unambiguous. This is clearly indicated from the higher prevalence of blindness in the poorer countries of the world compared with the developed. In addition, data also suggest that, depending on region, i.e. country, those with lower socioeconomic status are more likely to suffer from blindness around the world (18).

Numerous studies have examined the emotional impact exerted by vision loss. From a study by Apollonio et al. (19) the finding emerged that, in a sample of 1000 elderly people with severe visual impairment, the most depressed subjects with the least socialization and highest mortality rate were those in whom visual impairment had been neglected or not sufficiently corrected.

One typical patient reaction is depression of varying duration and severity, according to the patient’s underlying personal characteristics and socioeconomic status. A radical change in lifestyle has been indicated in all patients, including loss of employment, self-sufficiency and self-esteem. In some cases, this reaction is complicated to the point of precipitating the suicide.

There are three types of responses to sight loss: acceptance, denial and depression/anxiety. Acceptance of blindness is achieved through a physiological depressive reaction, which should be encouraged, because it has a cathartic effect (20).

A strong discrepancy emerged between the patients with different clinical prognoses. The psychopathological picture was worse for those with partial sight loss who displayed a more marked presence of depressed mood, anger and hostility (21). Correction of gradually deteriorating sight seemed to pose greater problems than adaptation to total, definite loss.

In a sample of patients with acquired blindness, Fitzgerland (22) reported the presence of depressed mood in 90% of the cases, accompanied by insomnia, loss of appetite, social withdrawal, loss of self-esteem, crying and suicidal ideation. The situation worsens if the psychopathological symptoms become chronic. In a 4-year follow-up study, he reported the persistence of a depressive-anxious syndrome in over 50% of the cases, indicating that the initial crisis had not been resolved (23). Psychotic symptoms persisted without any improvement after the 4 years, in patients displaying them at the onset of blindness.

Personal characteristics of individuals appear to be risk factors for the onset of depressive-anxious syndrome. Gentle, timid, obedient, conforming individuals, who respect authority, appeared to be most vulnerable. On the other hand, dependent personalities constitute an impediment to the development of an alternative lifestyle (24).

Being young, of good financial status, and in a moderate-to-high sociocultural level (25) have all proved to be protective factors against the onset of psychopathology, because these persons maintained good social relations and avoided isolation, which is a risk factor for depression. In addition, the subjects with a history of chronic organic pathologies appeared to have poorer coping skills with respect to their blindness, which was experienced as an additional discriminating factor and an attack on their person (25).

Another study revealed that no differences emerged in the process of accepting blindness between patients gradually becoming blind (over a few months period) and those who had a progressive impairment of their sight over several years. This means that the reaction to loss was the same regardless the duration of the process and that the handicap is more important than the time factor (25). Another important point of reference for patients with impairment of VA is family. Four possible reactions are described in family members: denial, refusal, acceptance and overprotection. The latter reaction is the most frequent, but also the most counterproductive, as it reinforces the patient’s objective physical and financial dependence on others. Dependence and loss of autonomy have been reported to result in self-depreciation (26). De Leo et al. (27) suggested that the foreseeable loss of sight can induce severe psychopathological distress that can lead to suicide. Another point of interest from this study was that sight restoration has also been directly associated with the onset of a psychopathological syndrome, even to the point of precipitating the suicide. When sight is restored, they must develop an understanding of a new environment, where things are perceived synchronically versus sequentially, often inducing shock to the patients. According to the conclusions of the same study, these reactions are a mirror image of the same trauma: a change in the individual’s lifestyle. By consequence, the onset in some patients of a more severe psychopathological syndrome often caused suicide to be considered a solution to the distress.

Many years ago research documents that Age-related macular degeneration (AMD) is associated with significant psychological distress and reduced function, comparable to that of other serious chronic illnesses. Furthermore, patients with heterogeneous eye diseases, when referred to a low-vision clinic reported high levels of depression and depressed low vision elderly were found to have disability independent of vision-related limitations. Untreated depression has been linked to worsen functioning (disability), immuno-endocrine dysregulation, greater likelihood of institutionalization and increased mortality (28).

An interesting randomized clinical trial used measures of depression, measures of disability, measure of vision and measure of demographic and health characteristics and comorbidity, to examine the prevalence of depressive disorders in community-dwelling adults with advance AMD and to find possible relationships in this population between depression, VA, the number of comorbid medical conditions and disability. In this study population, 32.5% were found to have a depressive disorder. Also high levels of disability were found. The correlation between depression and disability was very strong. One possible contribution to the strong relationship between depression and disability is that they are both related constructs. In this population, weaker association was found between VA and disability than between depression and disability. VA had little correlation with the severity of depressive symptoms. This suggests that depression may occur earlier in the course of ARMD (29). The search between the possibilities that comorbidity or VA added to the prediction of disability, showed that the number of comorbid conditions added little to the prediction of vision-specific disability. Finally, a randomized study examined the effectiveness of an AMD self-management program to improve quality of life as shown by measures of mood and function. The self-management group showed significant improvement in measures of mood and function compared with controls. Decreased emotional distress was associated with increased self-efficacy, while improvements in function were associated with increases in self-efficacy and perceived social support (30).

## CONCLUSIONS

Seeing the surrounding world is undoubtedly a wonderful experience. Throughout history, deprivation of eyesight has been perceived as the most severe form of punishment, second only to loss of life.

The findings emerging from studies on the subject clearly stress the need for greater sensitization to the problem, together with the establishment of guidelines for preventing the onset of secondary depression and suicidal behavior. The latter phenomenon, which is rare but not sporadic, is probably the most feared and most traumatic for attendant doctors, since it may in some cases represent a therapeutic defeat. Because recent treatments for depression are often effective and have few side-effects, ophthalmologists should consider referral for treatment of depression in blind patients. For this reason low vision rehabilitation, including cognitive behavioral therapy should be more readily available and recommended (31). In addition, self-management program offers an encouraging therapeutic addition to current medical treatments for an ever-growing aging population afflicted with AMD particularly in those who were initially depressed and seems suitable for an eye clinic setting.

We believe that the close cooperation between ophthalmologists and mental health professionals is the key to helping low vision patients.
